# MHS-VIT: Mamba hybrid self-attention vision transformers for traffic image detection

**DOI:** 10.1371/journal.pone.0325962

**Published:** 2025-06-30

**Authors:** Xude Zhang, Weihua Ou, Xiaoping Wu, Changzhen Zhang

**Affiliations:** 1 Engineering Research Center of Micro-Nano and Intelligent Manufacturing, Ministry of Education, Kaili University, Kaili, Guizhou, China; 2 College of Microelectronics and Artificial Intelligence, Kaili University, Kaili, Guizhou, China; 3 School of Big Data and Computer Science, Guizhou Normal University, Guiyang, Guizhou, China; Prince Mohammad Bin Fahd University, SAUDI ARABIA

## Abstract

With the rapid development of intelligent transportation systems, especially in traffic image detection tasks, the introduction of the transformer architecture greatly promotes the improvement of model performance. However, traditional transformer models have high computational costs during training and deployment due to the quadratic complexity of their self-attention mechanism, which limits their application in resource-constrained environments. To overcome this limitation, this paper proposes a novel hybrid architecture, Mamba Hybrid Self-Attention Vision Transformers (MHS-VIT), which combines the advantages of Mamba state-space model (SSM) and transformer to improve the modeling efficiency and performance of visual tasks and to enhance the modeling efficiency and accuracy of the model in processing traffic images. Mamba, as a linear time complexity SSM, can effectively reduce the computational burden without sacrificing performance. The self-attention mechanism of the transformer is good at capturing long-distance spatial dependencies in images, which is crucial for understanding complex traffic scenes. Experimental results showed that MHS-VIT exhibited excellent performances in traffic image detection tasks. Whether it is vehicle detection, pedestrian detection, or traffic sign recognition tasks, this model could accurately and quickly identify target objects. Compared with backbone networks of the same scale, MHS-VIT achieved significant improvements in accuracy and model parameter quantity.

## Introduction

With the acceleration of urbanization and the vigorous development of the automotive industry, the complexity and challenges of the transportation system, as the lifeline of urban operation, are becoming increasingly prominent. However, traditional traffic image detection methods often rely on manually designed features and rules, which are difficult to adapt to complex and changing traffic environments and constantly changing traffic demands [1]. For complex traffic images, continuous tracking of object trajectory can be achieved through Mamba time series modeling; Adding self-attention can realize real-time capture of relative position changes between vehicles, and enhance the response speed to emergencies; The local texture of multi-scale traffic image is extracted by self-attention at the low level, and the global semantics is integrated by Mamba at the high level to adjust the state transition matrix, give priority to the key objects and suppress the background noise; When the image is under the influence of complex illumination, the continuous state transfer based on Mamba has a smoothing effect on local illumination changes and reduces noise sensitivity. The self-attention module can dynamically strengthen the illumination invariant features and weaken the illumination related pixels; When it is necessary to process high frame rate video stream with low delay, Mamba’s parallel scanning algorithm makes full use of GPU parallelism and achieves higher throughput than other methods. Therefore, Mamba hybrid self-attention vision transformers play an indispensable role in traffic images.

In recent years, the rapid development of deep learning technology, especially its widespread application in the field of computer vision, has yielded unprecedented visual understanding and processing capabilities. Models have evolved from the classic convolutional neural network (CNN) [2–4] to the revolutionary transformer [5–8] model and then to the innovative Mamba architecture [9–12]. However, the limitation of CNN is their receptive field, where each neuron can only perceive a small area in the input image. In the field of object detection, transformers [13–15] have successfully solved the problem of the CNN receptive field limitation with their global modeling ability. The detection transformer (DETR) [16–19] series of models use transformers’ self-attention mechanism to transform object detection into an end-to-end sequence prediction problem, thereby achieving accurate localization and classification of objects in images. However, transformer models also face challenges such as high computational complexities and high memory consumption. The Mamba model was recently developed, which introduces a selective state-space model (SSM) combined with the recurrent framework of recurrent neural networks (RNNs) [20–22] and the parallel computing capability of transformers while maintaining the linear characteristics of the SSM [23]. Mamba can selectively propagate or forget information related to the sequence length dimension based on the current label, thereby improving its modeling ability for discrete modalities. Mamba achieves efficient computational performance through hardware-aware parallel algorithms, has linear scalability with the sequence length, and is capable of processing sequences up to millions in length.

In this article, we propose a traffic image detection model called Mamba Hybrid Self-Attention Vision Transformers (MHS-VIT), which combines the advantages of the Mamba model and vision transformers (ViT). The overall architecture of the model adopts a layered design, aiming to achieve accurate detection of targets in traffic images through multi-stage feature extraction and global context capture. In the field of deep learning, especially when processing image data, SSMs are mainly used to capture dependencies in time series or text sequences, but they lack effective modeling capabilities for the spatial structures and multi-channel features of image data. To compensate for this deficiency, we innovatively propose SVT Block and DLS Block components to provide better adaptability and improve the performance of image processing. The SVT Block can focus on each pixel in the input image, calculate their correlation, capture long-distance spatial dependencies in the image, and enhance the model’s ability to capture global information. The DLS Block uses dot multiplication combined with high-dimensional expressions to enhance the correlation between channels, which can enhance the interactions between channels by adjusting the eigenvalues on each channel without changing the dimensionality of the data space. MHS-VIT aims to build a new backbone network that combines the advantages of Mamba and vision transformers. This architecture, which uses a state-space transformation model, was applied for traffic image detection, effectively capturing global dependencies and using the strength of local convolution to improve the detection accuracy and model understanding of complex scenes. In addition, plug-and-play SVT blocks and DLS blocks can be integrated into deep neural networks to achieve effective image detection. The main contributions of this article can be summarized as follows:

- We propose the SSM-based MHS-VIT model to establish a new baseline for object detection in traffic images, laying a solid foundation for future development of more efficient image detection based on SSM.
- We propose the SVT Block, which can capture global information and long-range dependencies between pixels in images to compensate for the local modeling ability of SSM. Combined with residual connections, the input information is directly propagated across layers, simplifying the learning task of the network and making the learning of deep networks more efficient and stable.
- We propose the DLS Block to increase the complexity of the data in the spatial dimension and improve the model’s ability to capture complex features by optimizing the information flow and interactions between channels.
- A traffic image dataset was used for comprehensive experiments. The results showed that our MHS-VIT model has strong competitiveness compared with state-of-the-art CNN and transformer-based methods.

## Related work

CNNs performs well in image feature extraction, while Transformer achieves global context awareness through self attention mechanism. Mamba improves the processing capability of long sequence data through selective mechanisms and hardware aware algorithms. In this section, we attempt to propose a new method to further improve the performance and efficiency of traffic image detection.

### Convolutional neural network-based image detection

CNNs are a key technology in the field of computer vision. The powerful feature extraction ability of CNN is used to achieve automatic recognition and localization of target objects in images. R-CNN [24] first use a selective search algorithm to generate candidate regions, then use a CNN to extract features for each candidate region, and finally use a support vector machine classifier and bounding box regressor for object classification and localization. However, R-CNN suffer from a high computational complexity and slow detection speeds, as they perform independent CNN feature extraction on each candidate region. To improve the speed of R-CNN, the Fast R-CNN model [25] uses a region-of-interest pooling layer, which allows the entire image to pass through the CNN only once and then extracts the features of the candidate regions on the feature map. This improvement significantly improves the detection speed and maintains a high detection accuracy. Faster R-CNN [26] further introduces a region proposal network (RPN) to generate high-quality candidate regions, thereby further improving the detection speed and accuracy. The RPN and the detection network share convolutional features, achieving end-to-end training.

YOLOv1 [27], as the pioneering work of the YOLO series, detects all objects in the image through a single forward propagation, divides the image into grids, and predicts bounding boxes and categories for each grid. YOLOv2 [28] introduces anchor boxes and batch normalization to improve the detection accuracy and speed for small targets. YOLOv3 [29] adopts multi-scale feature extraction, improves the network structure, uses Darknet-53 as the backbone network, and introduces a feature pyramid network (FPN) to further enhance the detection performance. YOLOv4 [30] improves the detection accuracy by introducing various technologies while maintaining its speed advantage, but the introduction of these technologies correspondingly increases the demand for computing resources. YOLOv5 and subsequent versions [31–35] continue to seek a balance between speed and accuracy, introducing new network architectures [36] and optimization strategies to meet the needs of different application scenarios [37]. A CNN can automatically learn and extract effective feature representations from raw images, avoiding the tedious process of manually designing features in traditional methods. It can achieve certain robustness to image translation, rotation, scaling, and other transformations and can adapt to object detection tasks in different scenarios. However, CNNs mainly focus on local features of images, and they may not perform well in tasks that require global information. When recognizing objects, a CNN may not fully consider the relative spatial direction and hierarchical structure between features, leading to misjudgments in some cases.

### Transformer-based image detection

Transformer-based object detection has been an important research direction in the field of computer vision in recent years. The powerful global context modeling and parallel computing capabilities of transformer models have introduced new ideas for object detection tasks. DETR [38] is an object detection model based on transformers, which achieves end-to-end training through the encoder–decoder architecture of transformers. The key to the DETR model lies in its unique training strategy and object matching mechanism. It optimizes the loss function through a bipartite graph matching algorithm to improve the accuracy and robustness of detection. The deformable DETR [39] introduces a deformable attention mechanism to reduce the complexity of self-attention computations. Through a multi-scale deformable attention module, it achieves accurate detection of objects of different scales in images. RT-DETR [16] includes an efficient hybrid encoder that decouples an attention-based intrascale feature interaction (AIFI) module and a CNN-based cross-scale feature fusion module (CCFM) to efficiently process multi-scale features, reduce the computational costs, and enable the model to achieve real-time detection while maintaining high accuracy. However, transformer models have numerous parameters and complex structures, requiring large-scale datasets and powerful computing resources for training and optimization. Small target images occupy fewer pixels and provide limited feature information, resulting in certain limitations of transformer-based models in small target detection.

### Mamba-based image detection

Recently, the SSM [23,40,41] has played an important role in the research of artificial intelligence. Its unique modeling ability and efficient computational characteristics make it an important tool for processing complex dynamic systems, time series data, and long series data. In this context, Mamba [9], as an innovative architecture based on SSM, has attracted considerable attention for its linear complexity and efficient solution to the computational efficiency problems faced by transformers when processing long sequences. Vision Mamba [11] is a pure visual backbone model design based on an SSM, which introduces a cross-scan module (CSM) to achieve selective scanning of two-dimensional images, thereby enhancing the model’s ability to process image data. Specifically, the cross-scanning module can scan images in different directions, extract richer feature information, and model and analyze them through the SSM. This design enables Vision Mamba to effectively capture global contextual information and local detail features in images without the need for additional self-attention mechanisms. VMamba [10] introduces a selective scanning mechanism to reduce the quadratic complexity of traditional attention computations to linear while retaining the advantage of a global receptive field. Additionally, a CSM is introduced to solve the problem of directional sensitivity in two-dimensional image scanning and achieve efficient extraction of multi-scale features. Applying Mamba to object detection tasks can effectively improve the detection performance. Through its bidirectional scanning mechanism and global modeling capability, Mamba can more accurately capture target features in images and reduce the interference of background noise. This helps to improve the accuracy and robustness of object detection, enabling the model to maintain a good performance even in complex scenarios. Mamba provides strong support for object detection tasks with its efficient modeling ability and computational efficiency [42]. In the future, with the continuous development of technology and the expansion of application scenarios, Mamba’s application prospects in the field of object detection will be even broader.

## Method

### Preliminaries

The self-attention mechanism of the standard transformer can be formalized as:

$$Attention\left(Q,K,\;V\right)=softmax\left(\frac{{QK}^{T}}{\sqrt{d_{k}}}\right)V$$ (1)

where $Q,K,V\in R^{N\times d}$, computational complexity $O\left(N^{2}d\right)$, When the sequence length n is large, the complexity of quadratic will lead to huge consumption of computing resources and low computational efficiency.

In this section, we briefly introduce the fundamentals of SSM [23], The SSM-based structured state-space sequence model S4 and Mamba are derived from a continuous system that maps a univariate sequence x(t)∈R to an output sequence y(t) via an implicit latent intermediate state $h\left(t\right)\in R^{N}$. The mathematical definition of the system can be summarized as follows:

$$h^{\prime }\left(t\right)=Ah\left(t\right)+Bx\left(t\right)$$ (2)

y(t)=Ch(t) (3)

where $A\in R^{N\times N}$ represents the state matrix, and $B\in R^{N\times 1}$ and $C\in R^{N\times 1}$ represent the projection parameters, computational complexity O(N).

The Mamba model cleverly introduces a key time scale parameter Δ when discretizing the continuous SSM to adapt to deep learning scenarios. The parameter represents the time step in the discretization process and determines the level of refinement when the continuous dynamics are approximated as discrete sequences. To effectively achieve discretization, Mamba uses a zero-order keeper to convert A and B into discrete parameters $\overset{―}{A}$ and $\overset{―}{B}$, respectively, using fixed discretization rules, defined as follows:

$$\overset{―}{A}=\exp\left(\Delta A\right)$$ (4)

$$\overset{―}{B}={\left(\Delta A\right)}^{-1}\left(\exp\left(\Delta A\right)-I\right)\Delta B$$ (5)

where ΔA and ΔB represent the discrete correspondence of continuous parameters in a given time interval, respectively, and I represents the identity matrix.

Mamba’s linear complexity makes it superior to transformer in computational efficiency and memory usage, especially when dealing with long sequences of traffic scene data. At the same time, through the state space model, Mamba can effectively capture the spatio-temporal correlation of traffic data and improve the detection ability of the model for traffic scenes.

### Overall architecture

**Fig 1:**
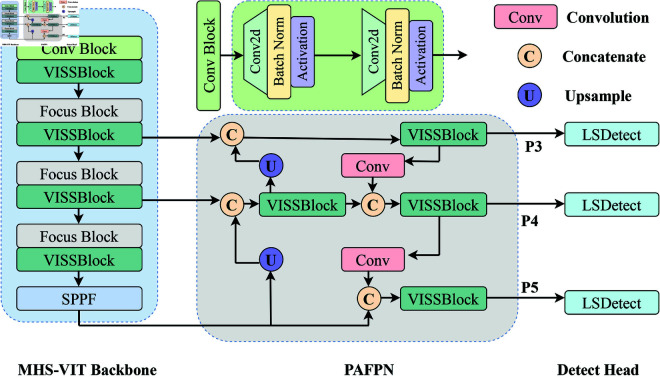
Illustration of the Mamba Hybrid Self-Attention Vision Transformers (MHS-VIT) architecture.

In this section, we mainly introduce the structure of MHS-VIT, as shown in Fig 1. The model consists of the MHS-VIT Backbone, PAFPN, and Detect Head. The MHS-VIT Backbone is composed of Conv Block and Downsample Block components for feature extraction, while the PAFPN is mainly composed of VISSBlock, Concatenate, and Upsample components. Multi-scale feature fusion helps the model better capture the details and contextual relationships of the target, thereby improving the accuracy of detection. The Detect Head component is responsible for predicting the bounding boxes, categories, and confidence levels of targets in images.

### Focus block

**Fig 2:**
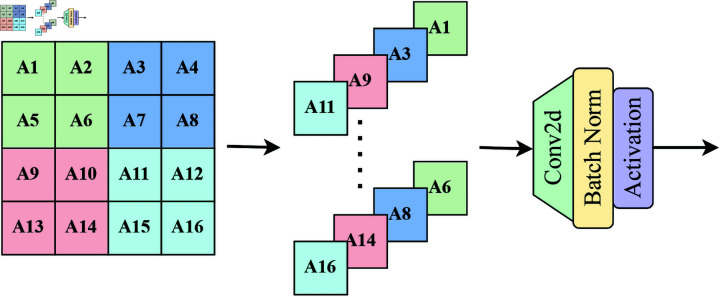
Illustration of the Focus Block architecture.

Focus Block, as shown in Fig 2, cuts the input tensor along specific dimensions to form multiple smaller tensor fragments, which are then rearranged and stacked together to form tensors with richer information. By changing the arrangement of features, additional spatial- or channel-level information interactions are introduced, which can enhance the model’s perception ability and make it easier to capture key feature information, thereby improving the accuracy and robustness of detection.

Focus Block provides a new perspective for the learning process of the model by changing the presentation of data, which helps the model learn effective feature representations more easily during training and accelerates the convergence process. As the Focus Block module can enhance the model’s feature extraction ability, it can improve the model’s generalization ability to a certain extent, enabling the model to perform better when facing new and unseen data.

### VISSBlock

**Fig 3:**
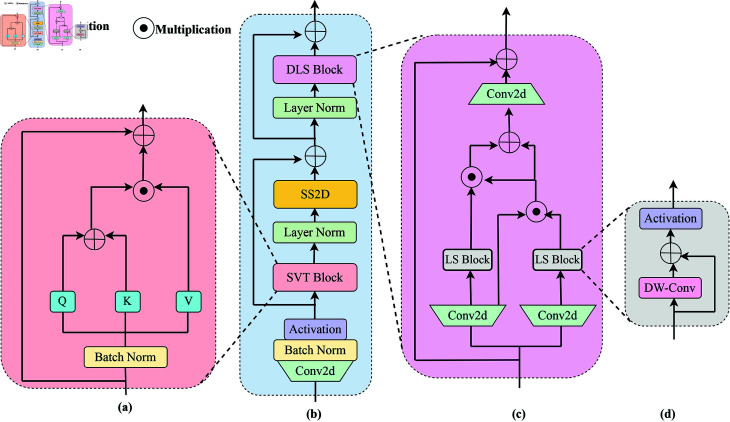
(a) Detailed structure of the SVT Block. (b) Illustration of the VISSBlock architecture. (c) Illustration of the DLS Block. (d) Illustration of the LS Block.

As shown in Fig 3, VISSBlock is the core of MHS-VIT, playing a key role in improving the model’s feature extraction capability and maintaining a high processing efficiency and throughput. VISSBlock introduces the 2D-Selective-Scan for Vision Data (SS2D) model using a four-way scanning strategy, scanning simultaneously from all four corners of the feature map to ensure that each element in the feature can integrate information from all other positions in different directions, achieving efficient extraction of multi-scale features. This not only preserves the spatial structure information of the image but also enhances the model’s ability to capture image details by scanning in different directions. The SS2D module also merges multiple one-dimensional vectors into a two-dimensional feature output through a scanning merging step, further improving the compactness and effectiveness of the feature representation.

An SVT Block component based on convolutional additive self-attention is added [43]. Q and K are transformed by mapping the channel attention and spatial attention to construct an additive similarity function to calculate the similarity between queries and keys. This similarity function is based on the output activated by a sigmoid and implemented through a convolution operation, preserving the original feature dimension while avoiding information loss. Finally, the result of the similarity function is weighted and summed with V to obtain the final output, which contains global context information and has a low computational complexity.

The input feature information of the DLS Block is subjected to convolution and LS Block for feature extraction. The LS Block consists of a depthwise separable convolution, residual module, and activation function. The output feature information is effectively enhanced by combining dot multiplication and a high-dimensional expression, which can effectively enhance the correlation between channels without changing the dimension of the data space.

### LSDetect

**Fig 4:**
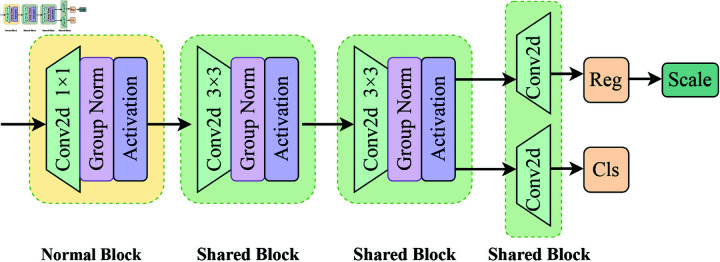
Illustration of the LSDetect architecture.

As shown in Fig 4, LSDetect is the detection head part of MHS-VIT, consisting of a convolution, GroupNorm, and an activation function. The detection head needs to go through a common module and a shared module. The common module has different parameters for the three types of detection heads during feature extraction, while the shared module has the same parameters for the three types of detection heads. Using GroupNorm can improve the performance of the detection head localization and classification. By using shared convolution, the number of parameters can be significantly reduced, and the model becomes lightweight. To address the issue of inconsistent target scales detected by each detection head while using shared convolution, a Scale layer is used to scale the features.

## Experiments

We conducted comprehensive experiments on traffic image detection tasks. Specifically, we evaluated the performance of MHS-VIT in image detection tasks on signal light datasets and traffic datasets. In terms of the experimental environment, we selected Ubuntu 20.04, which is stable and widely supported, as the cornerstone operating system to ensure compatibility and repeatability of the experimental environment. To fully tap into the computational potential of the model, we employed an NVIDIA RTX 4090D GPU, whose powerful computing power combined with CUDA 11.8 optimization provides a solid hardware foundation for processing high-resolution and high-complexity traffic image data. Before model training, unify the width and height of all input images to 640,set the model hyperparameters epochs to 100, batch size of 8, use SGD for optimization with an initial learning rate of 1e–2 and a final learning rate of 1e–4, The warm-up epochs are 3, and the learning rate is adjusted using Cosine Annealing format, and finally save the model weights only when the validation set reaches the new optimal performance.

### Datasets

This study used the Traffic Road Object Dataset (TROD), which consists of 6633 images covering various traffic scenarios, including but not limited to urban roads, highways, rural roads, and complex intersections. Each image underwent strict screening and annotation to ensure the accuracy and reliability of the data. The objects in the dataset are divided into five categories: cycle, bus, car, motorbike, and person. Considering the dynamic changes in the transportation environment, the TROD aims to include images from different periods and weather conditions to simulate complex real-world situations. The dataset can be further expanded, and researchers can add new images and annotation information as needed to meet different research needs.

With the rapid development of autonomous driving technology, the detection and recognition of traffic signals have become an important component of the perception systems of autonomous vehicles. Accurately and quickly identifying the status of traffic signals is crucial for vehicles to make correct driving decisions. This study used the Traffic Signal Light Dataset (TSLD), which consists of 4564 images with four categories: red, green, off, and yellow. The TSLD contains various types of traffic signal light images, including images of different shapes, colors, and lighting conditions, which help to train more robust recognition models. By training recognition models based on the TSLD, autonomous vehicles can accurately recognize the status of traffic signals and make corresponding driving decisions according to traffic rules.

### Baseline methods

To verify the effectiveness of the proposed method, we chose Mamba YOLO as our baseline model. MHS-VIT has the same variant as Mamba YOLO, namely T/B/L, and all the models were trained under the same hyperparameter configuration, with the same number of training iterations and batch size. MHS-VIT was directly compared with other benchmark methods (YOLOv5 [32], YOLOv6 [34], YOLOv8 [33], YOLOv10 [35], and MambaYOLO [42]) to observe and evaluate metrics to determine the performance of the model.

In traffic image detection, the selection of evaluation indicators not only reflects the accuracy of model detection but also reveals the performance of the model in terms of detail capture, robustness, and sensitivity to outliers. When evaluating the performance of MHS-VIT and the baseline methods, to measure the detection performance, we chose parameters including the accuracy, recall, and mean average precision (mAP), with higher values indicating a better performance.

### Ablation study on MHS-VIT

We independently examined each module in VISSBlock to evaluate its effect on traffic image detection and conducted ablation experiments on the TROD using MHS-VIT. Table 1 shows the effect of the SVT Block, DLS Block, and LS Block components on the image detection process. YES indicates that the module was used, and NO indicates that the module was not used. The ablation experiment was evaluated using the precision, recall, and mAP, and the higher the values were, the better the detection effect was.Through the comparison of MHS-VIT ablation research results, it is obvious that using model 6(our) is almost the best in precision, recall, mAP50 and mAP50-95.

**Table 1:** Ablation study on MHS-VIT.

Model	SVT Block	DLS Block	LS Block	Precision (%)	Recall (%)	mAP50 (%)	mAP50-95 (%)
1	NO	NO	NO	88.8	84.5	91.5	67.4
2	NO	**YES**	**YES**	90.0	87.5	92.8	69.5
3	NO	**YES**	NO	89.8	86.5	91.5	68.6
4	**YES**	NO	NO	89.9	85.3	91.9	67.9
5	**YES**	**YES**	NO	90.0	85.5	92.2	69.2
6	**YES**	**YES**	**YES**	89.9	87.7	93.0	71.7

**Fig 5:**
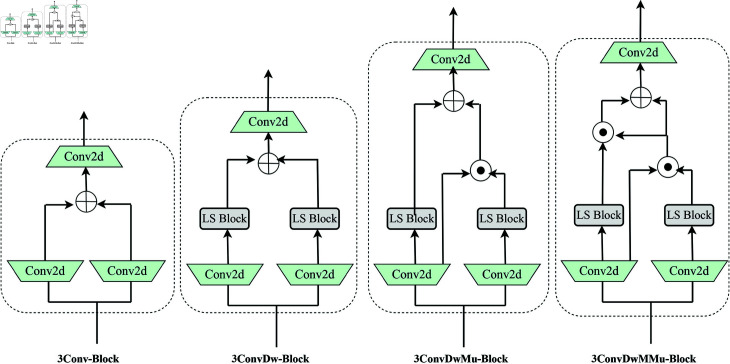
DLS Block integration designs explored in the ablation study.

Evaluate each module in the DLS Block Fig 5 to perform ablation experiments on the TROD dataset. Table 2 shows the impact of 3Conv-Block, 3ConvDw-Block, 3ConvDwMu-Block, 3ConvDwMMu-Block and DLS Block(our) on traffic image detection. Ablation experiments were evaluated by precision, recall and map. The higher the value, the better the detection effect.Through the comparison of DLS Block ablation research results, it is obvious that using DLS Block(our) is the best in precision, recall, mAP50 and mAP50-95.

**Table 2:** Ablation study on DLS block

Model	Precision (%)	Recall (%)	mAP50 (%)	mAP50-95 (%)
3Conv-Block	86.2	83.4	89.8	65.6
3ConvDw-Block	87.5	84.5	90.2	66.7
3ConvDwMu-Block	88.6	85.3	90.8	67.3
3ConvDwMMu-Block	89.1	85.6	91.2	67.8
**DLS Block(our)**	**89.9**	**87.7**	**93.0**	**71.7**

### Quantitative comparisons with state-of-the-art image detection methods

**Fig 6:**
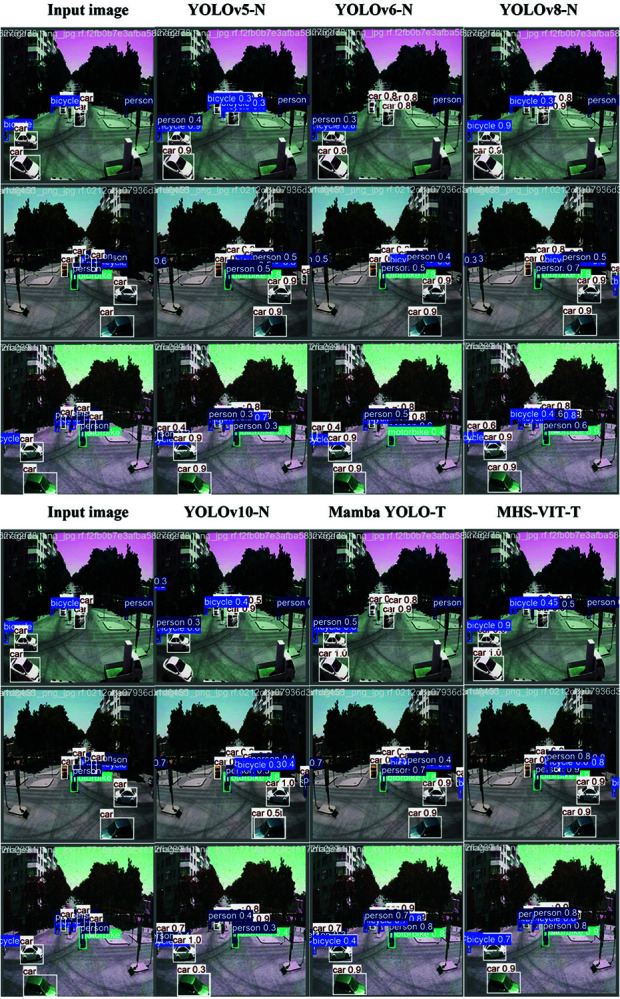
Comparison chart of different model detection results on the TROD datasets.

The comparison of the results from MHS-VIT and the other image detection models on the TROD, where the proposed method was compared based on various evaluation metrics, including the precision, recall, mAP, floating point operations per second (FLOPs) (G), and Par. (M). The performance of MHS-VIT on the TROD was almost superior to all reference models, Fig 6 shows the comparison of detection results for different models on the TROD datasets.

**Fig 7:**
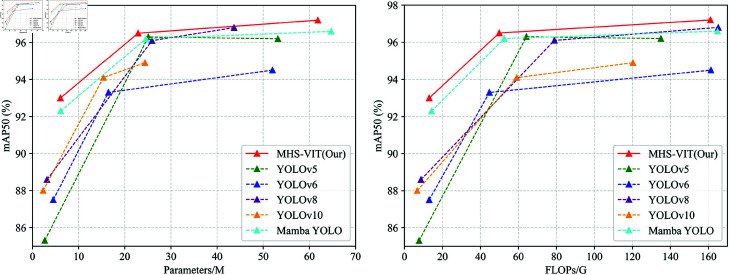
Compare the performance of different model on the TROD dataset.

As shown in Table 3, our MHS-VIT achieved state-of-the-art performances at various model scales. We first compared MHS-VIT with the baseline model, Mamba YOLO. With the T/B/L variants, our MHS-VIT achieved 0.7%/0.3%/0.6% improvements in mAP50, respectively, with improvements of 0.9%/0.6%/1.6% in mAP50-95, parameter reductions of 1.1%/7%/4.5%, and computational reductions of 9.1%/5%/2.3%, respectively. Compared with other YOLOs, MHS-VIT also demonstrated a superior trade-off between the accuracy and the computational cost. Compared with YOLOv5-N/YOLOv6-N/YOLOv8-N/YOLOv10-N, MHS-VIT-T improved the mAP50 by 7.7%/5.5%/4.4%/5% and the mAP50-95 by 10.6%/7.3%/6.4%/8.4%, respectively. Compared with YOLOv5-M/YOLOv6-M/YOLOv8-M/YOLOv10-M, MHS-VIT-B achieved mAP50 improvements of 0.2%/2%/0.4%/2.5%, respectively; mAP50-95 improvements of 0.5%/2.4%/1.2%/5.2%, respectively; parameter reductions of 8.8%/56%/11.7%/-48.8%, respectively; and computation reductions of 22.3%/69%/36.8%/15.5%. Compared with YOLOv5-L/YOLOv6-L/YOLOv8-L/YOLOv10-L, MHS-VIT-L achieved 1%/2.3%/0.4%2.4% improvements in mAP50 and improvements of 1.7%/3%/1.8%7.2% in mAP50-95, respectively. These comparison results indicate that the proposed model provided significant improvements in MHS-VIT at different scales compared with existing state-of-the-art methods.Compare the performance of different model on the TROD dataset,The Fig 7 left figure shows that MHS-VIT(our) performs best under the same Parameters, and the right figure shows that MHS-VIT(our) performs best under the same FLOPs.

**Table 3:** Comparison of MHS-VIT with other image detection networks on the TROD (bold indicates our framework).

Framework	Precision (%)	Recall (%)	mAP50 (%)	mAP50-95 (%)	FLOPs (G)	Par (M)
YOLOv5-N [32]	81.8	83.1	85.3	61.1	7.7	2.65
YOLOv6-N [34]	87.4	82.8	87.5	64.4	13	4.5
YOLOv8-N [33]	85.3	85.1	88.6	65.3	8.7	3.15
YOLOv10-N [35]	85.3	79.7	88	63.3	6.7	2.29
Mamba YOLO-T [42]	90.2	86.6	92.3	70.8	14.3	6.13
**MHS-VIT-T(Our)**	**89.9**	**87.7**	**93**	**71.7**	**13**	**6.0**6
YOLOv5-M [32]	93.9	92	96.3	79	64.2	25.09
YOLOv6-M [34]	93.7	90.3	94.5	77.5	161.3	52
YOLOv8-M [33]	92.8	92.7	96.1	78.4	78.9	25.89
YOLOv10-M [35]	92.1	91.4	94.1	75.2	59.1	15.36
Mamba YOLO-B [42]	93	92.2	96.2	78.8	52.5	24.58
**MHS-VIT-B(Our)**	**93.9**	**91.7**	**96.5**	**79.4**	**49.9**	**22.87**
YOLOv5-L [32]	93	93.1	96.2	79.9	135	53.19
YOLOv6-L [34]	93.4	90.4	94.9	78.9	391.4	110.89
YOLOv8-L [33]	93.2	93	96.8	79.8	165.2	43.67
YOLOv10-L [35]	91.4	90.8	94.9	75.8	120.3	24.37
Mamba YOLO-L [42]	93.1	92.7	96.6	79.7	164.8	64.73
**MHS-VIT-L(Our)**	**94.4**	**93.9**	**97.2**	**81.3**	**161.1**	**61.83**

**Fig 8:**
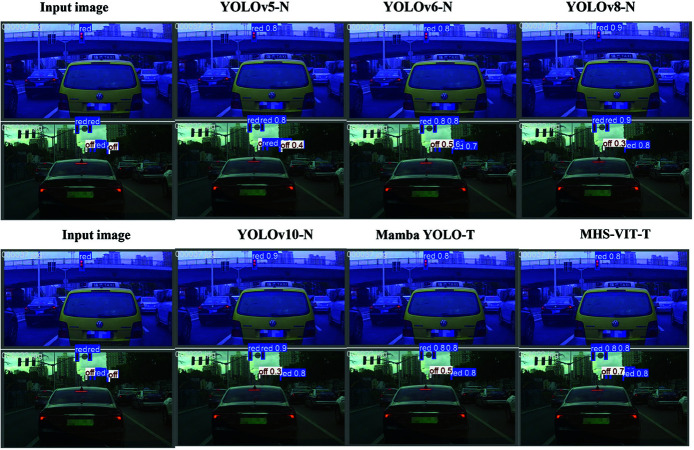
Comparison chart of different model detection results on the TSLD datasets.

**Fig 9:**
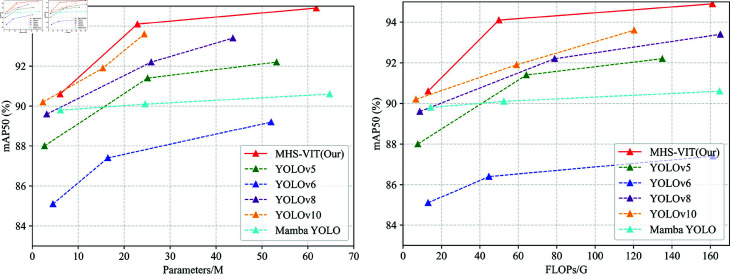
Compare the performance of different model on the TSLD dataset.

Table 4 shows the comparison of the results between MHS-VIT and the other image detection models on the TSLD. Our MHS-VIT achieved state-of-the-art performances at various model scales. We first compared MHS-VIT with the baseline model, Mamba YOLO. With the T/B/L variants, our MHS-VIT achieved 0.8%/4%/4.3% improvements in mAP50, 0.2%/2%/2.9% improvements in mAP50-95, 1.1%/7%/4.5% reductions in the parameters, and 9.1%/5%/2.3% reductions in the computations, respectively. Compared to other YOLOs, MHS-VIT also demonstrated a superior trade-off between the accuracy and the computational cost. Compared with YOLOv5-N/YOLOv6-N/YOLOv8-N/YOLOv10-N, MHS-VIT-T improved the mAP50 by 2.9%/6.5%/1.1%/0.4% and the mAP50-95 by 4%/6.4%/3.6%/3.1%, respectively. Compared with YOLOv5-M/YOLOv6-M/YOLOv8-M/YOLOv10-M, MHS-VIT-B achieved mAP50 improvements of 3%/7.6%/2%/2.3%, mAP50-95 improvements of 1.7%/6.6%/3%/0.4%, parameter reductions of 8.8%/56%/11.7%/-48%, and computation reductions of 22.3%/69%/36.8%/15.5%, respectively. These comparison results indicate that the proposed model provided significant improvements over existing state-of-the-art methods at different scales of MHS-VIT. Fig 8 shows the comparison of detection results for different models on the TSLD datasets.Compare the performance of different model on the TSLD dataset, The Fig 9 left figure shows that MHS-VIT(our) performs best under the same Parameters, and the right figure shows that MHS-VIT(our) performs best under the same FLOPs.

**Table 4:** Comparison of MHS-VIT with other image detection networks on the TSLD (bold indicates our framework).

Framework	Precision (%)	Recall (%)	mAP50 (%)	mAP50-95 (%)	FLOPs (G)	Par (M)
YOLOv5-N [32]	90.1	83	88	57.3	7.7	2.65
YOLOv6-N [34]	82.3	84.7	85.1	56	13	4.5
YOLOv8-N [33]	91.5	86.1	89.6	57.5	8.7	3.15
YOLOv10-N [35]	83.1	84.4	90.2	57.8	6.7	2.29
Mamba YOLO-T [42]	90	85.5	89.8	59.4	14.3	6.13
**MHS-VIT-T(Our)**	**91.5**	**88.8**	**90.6**	**59.6**	**13**	**6.06**
YOLOv5-M [32]	91.5	89	91.4	61.3	64.2	25.09
YOLOv6-M [34]	87.5	79.7	87.4	58.5	161.3	52
YOLOv8-M [33]	89.8	88.3	92.2	60.5	78.9	25.89
YOLOv10-M [35]	88.9	89.2	91.9	62.1	59.1	15.36
Mamba YOLO-B [42]	87.8	85.2	90.1	60.4	52.5	24.58
**MHS-VIT-B(Our)**	**90.2**	**91.4**	**94.1**	**62.4**	**49.9**	**22.87**
YOLOv5-L [32]	92.6	89.2	92.2	62	135	53.19
YOLOv6-L [34]	93	81	89.2	58.6	391.4	110.89
YOLOv8-L [33]	89.8	89.7	93.4	63.5	165.2	43.67
YOLOv10-L [35]	91	91.1	93.6	62.3	120.3	24.37
Mamba YOLO-L [42]	88.2	86.3	90.6	61.3	164.8	64.73
**MHS-VIT-L(Our)**	**91.2**	**92.5**	**94.9**	**64.2**	**161.1**	**61.83**

## Conclusions

This article proposed a traffic image detection model called MHS-VIT. To solve the problems of the difficulty in capturing long-distance dependencies and the high ViT computational complexity in CNN global image information, the Mamba model was used in MHS-VIT, and an update mechanism for the state-space representation and linear time complexity was employed to enhance the model’s understanding of traffic scenes. It also enables the model to maintain efficient operation when processing datasets, reducing the computational complexity. Our MHS-VIT showed superiority over other methods on the TROD and TSLD. An ablation study also demonstrated that the proposed SVT Block and DLS Block components played crucial roles in improving the model performance and reducing the computational complexity. The proposed model provides new ideas and methods for research in the field of intelligent transportation. Its superior performance and efficient computational efficiency demonstrate the enormous potential of the model in practical applications. We look forward to continuing to explore the potential of the Mamba model in future research and contributing more to the construction of intelligent transportation systems.
